# Peer Review of “Utility of the ROX Index in Predicting Intubation for Patients With COVID-19–Related Hypoxemic Respiratory Failure Receiving High-Flow Nasal Therapy: Retrospective Cohort Study”

**DOI:** 10.2196/31896

**Published:** 2021-08-27

**Authors:** Mukul Sehgal

*This is a peer-review report submitted for the paper “Utility of the ROX Index in Predicting Intubation for Patients With COVID-19–Related Hypoxemic Respiratory Failure Receiving High-Flow Nasal Therapy: Retrospective Cohort Study”.*

## Round 1 Review

### General Comments

This paper [1] is an important resource for the use of high-flow nasal cannula in patients with COVID-19 and provides important insights on the current COVID-19 pandemic. Respiratory support for patients with COVID-19 is an important topic for critical patients.

### Specific Comments

The paper can be improved in certain areas like methods and design. Also, there is some duplication of the results in the discussion.

#### Major Comments

1. Inclusion criteria need to be well defined.
2. Initial HFNT (high-flow nasal therapy) settings: there is a discrepancy between the flow mentioned in the Method and Design section (35 L/min) and that mentioned in the Results section (33.5, SD 11.7 L/min of flow). Can you please clarify?
3. The discussion needs to be rewritten. It seems like a duplication of the results, especially the first paragraph.

#### Minor Comments

1. The dose of methylprednisolone should read “mg/kg.”
2. Screening criteria: were the patients identified with high clinical suspicion based on computed tomography COVID-19 negative? If yes, please mention that in the paper.
3. In the sentence, “At initiation of HFNC, a ROX of <5 was predictive of intubation (OR 2.137, *P*=.051),” what was the confidence interval? The *P* value is greater than .05.
4. In the sentence, “Any change in ROX of less than or equal to zero after HFNT initiation over 24 hours was also predictive of intubation,” what do you mean by change less than or equal to zero? This sentence is a little confusing.
5. There are multiple references of ROC=0.77 and ROC=0.86; did you mean AUC (area under the ROC curve) or C-statistic?
